# Angiomyolipome rénale: à propos de huit cas

**DOI:** 10.11604/pamj.2014.19.138.4349

**Published:** 2014-10-10

**Authors:** Mouad Statoua, Jihad El Ghanmi, Tarik Karmouni, Khalid El Khader, Abdellatif Koutani, Ahmed Iben Attya

**Affiliations:** 1 Service d'Urologie B, Centre Hospitalier Universitaire IBN Sina, Rabat, Maroc

**Keywords:** Angiomyolipome, scanner, histologie, embolisation, néphrectomie totale, néphrectomie partielle, Angiomyolipoma, CT scan, histology, embolization, total nephrectomy, partial nephrectomy

## Abstract

L'angiomyolipome est la tumeur bénigne la plus fréquente des masses dolides du rein, elle représente un cadre de fréquence de 1 à 3% des tumeurs du rein, sa composition histologique est faite de trois contingents: graisseux, fibres musculaires lisses et vasculaires a des proportions variables, elle sévit sur un cadre sporadique et peut s'exprimer dans un cadre congénitale comme manifestation de la sclérose tubéreuse de Bourneville. Nous rapportons l'expérience du service d'Urologie B du CHU IBN Sina de Rabat dans la prise en charge de huit cas d'angiomyolipome sur une période s’étalant sur six ans en précisant les manifestations cliniques, données de l'imagerie, le résultat histologique et la conduite thérapeutique. Les manifestations cliniques ne lui sont pas spécifiques, elle peut se manifester par des lombalgies, crise de colique néphritiques, hématurie, masse palpable dans le flanc. La TDM reste l'examen radiologique le plus sensible en mettant en évidence la présence de graisse au sein de la masse, l'histologie confirme le diagnostic et il n'y a aucun consensus qui régit la prise en charge de ce type de tumeur, on peut admettre une surveillance pour les petites masses de moins de 4 cm et traité les formes symptomatiques ou qui dépasse les 4 cm par embolisation ou chirurgie partielle ou totale. Les nouvelles thérapeutiques focales peuvent révolutionner la prise en charge de ce type de tumeur bénigne, mais les études sont toujours en cours.

## Introduction

L'AML est la tumeur rénale bénigne la plus fréquente 1 à 3% des tumeurs solides du rein [1], Cette lésion se compose dans des proportions variables, d′un contingent graisseux souvent le plus abondant, d′un contingent de cellules musculaires lisses et d′un contingent d′origine vasculaire [2, 3]. Elle peut se présenter sous forme sporadique (la plus fréquente) ou sous forme congénitale et rentre dans le cadre de la sclérose tubéreuse de Bourneville (STB). A travers se travail nous rapporterons l'expérience de notre service dans la prise en charge des AML ainsi que mettre le point sur les particularités épidémiologiques, diagnostiques, thérapeutiques et pronostiques de cette pathologie.

## Méthodes

Nous rapportons une étude rétrospective de huit cas d'AML rénaux colligés dans le service d'Urologie B du CHU IBN SINA rabat sur une période de 6 ans, de janvier 2008 à janvier 2014. Le diagnostic de présomption a été posé devant des arguments cliniques et radiologiques avant la confirmation histologique lorsqu'un geste chirurgical a été réalisé. Le Tableau 1 résume la somme des observations cliniques.

**Table 1:** Récapitulatif des principaux éléments cliniques et radiologiques des observations colligées

Cas	Sexe/Age (ans)	ATCD	CDD	Siège	Dimensions	Traitement
1	H/55	-	HRPS + Etat de choc HématurieLombalgies	Droit	92 mm	Néphrectomie droite d'hémostase
2	F/33	-	Lombalgies Absence d'hématurie	Droit	160 mm	NTE Droite pour suspicion de lésion tumorale maligne
3	H/24	STB	Hématurie Etat de chocLombalgies	Bilatéral	G : 54 mm D : 108 et 55 mm	Néphrectomie droite d'hémostase
4	F/32	STB	Hématurie Lombalgies	Bilatéral	G : 65 mm D : 88 mm	- EAS de L'AML Droit - Néphrectomie droite d'hémostase pour récidive hémorragique
5	F/61	SJPU	Asymptomatique	Droit	55 mm	Néphrectomie partielle supérieure droite associée à la cure du SJPU
6	H/54	-	Hématurie lombalgies	Gauche	109 mm	Néphrectomie gauche partielle
7	F/44	Cholécystite	Asymptomatique	Gauche	42 mm	Surveillance
8	H/28	STB	Lombalgies	Bilatéral	G : 60 mm D : 75 mm	EAS

Notre étude se compose de 8 patients avec un âge moyen de 41,6 ans (24 et 61 ans), répartis de façon égale entre les deux sexes soit 4 hommes pour 4 femmes et qui peuvent être sélectionné en deux groupes: Groupe 1 formé de 5 patients présentant une forme sporadique et isolée de l'AML (62,5%); Groupe 2 formé de 3 patients (37,5%) qui s'intègrent des la forme phacomatose héréditaire en l'occurrence la Sclérose Tubéreuse de Bourneville.

L’évaluation clinique a porté sur un interrogatoire recherchant les signes cliniques d'appels ainsi qu'un caractère familiale de l'affection, complété par un bilan biologique qui consistait en la réalisation d'une numération de formule sanguine pour apprécier le retentissement du saignement sur le taux d'hémoglobine ainsi que la réalisation d'un ionogramme sanguin pour apprécier la fonction rénale, une évaluation radiologique a porté sur la réalisation d'une échographie abdominale complété par un scanner abdomino-pelvien permettant de mieux caractériser les lésions. Au terme de tous ses bilans une attitude thérapeutique a été décidée et qui penchait vers une attitude conservatrice de surveillance ou une prise en charge chirurgicale.

## Résultats

Six cas de nos patients présentaient une symptomatologie clinique faite essentiellement de lombalgie unilatérale sourde et modérée ou une hématurie macroscopique caillotante conduisant les patients à consulter, deux des formes avec hématurie se sont présenté aux urgences dans un tableau d'hématurie foudroyante associé a un hématome rétropéritonéale avec instabilité hémodynamique nécessitant une prise en charge urgente. Les deux derniers cas la découverte été fortuite lors de la réalisation d'une échographie abdominale de routine pour le suivi d'un syndrome de jonction pyélourétérale chez une patiente et dans le cadre du bilan d'une cholécystite chez une autre.

**Figure 1:**
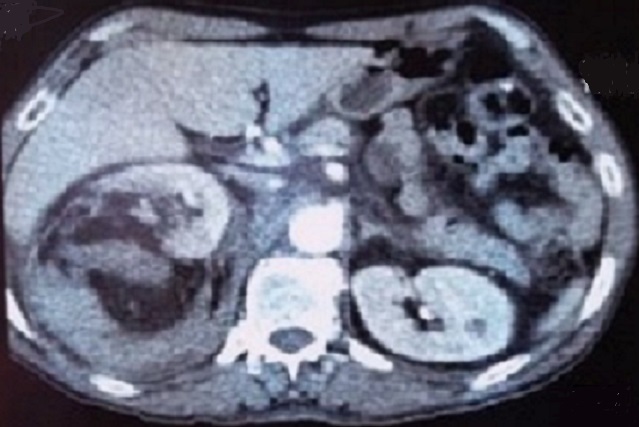
Angiomyolipome rénale polaire supérieur droit révélé par un hématome spontanée rétropéritonéale

**Figure 2:**
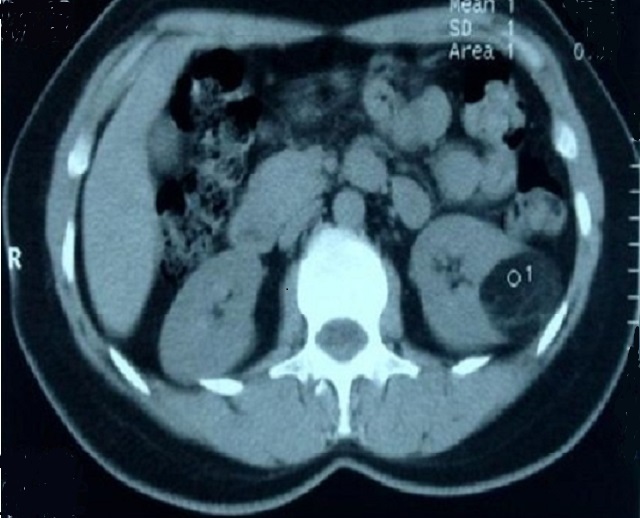
Angiomyolipome rénale gauche isolé de 42 mm chez une femme de 44 ans

Le bilan biologique a été réalisé pour apprécier le retentissement du saignement sur l’état général (NFS avec appréciation du taux d'hémoglobine et évaluation de la fonction rénale). Une échographie réalisée en première intention permettait d'orienter le diagnostic en mettant en évidence une masse hyperechogène et hétérogène, un complément scannographique a été réalisé à chaque fois permettant de poser le diagnostic présomptif d'AML par la découverte au sein de la masse rénale d'une composante graisseuse caractérisée par sa densité négative (Figure 1, Figure 2). Au total 12 AML ont été diagnostiqué dont 7 à droite et 5 à gauche (la bilatéralité des lésions a été observée chez les 3 patients porteurs de STB), la taille moyenne des masses était de 80,3mm (42à 160mm).

**Figure 3:**
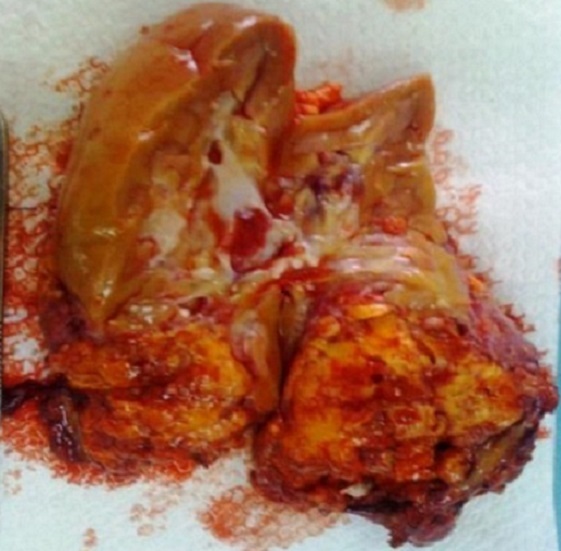
Aspect à la coupe après néphrectomie total droite

**Figure 4:**
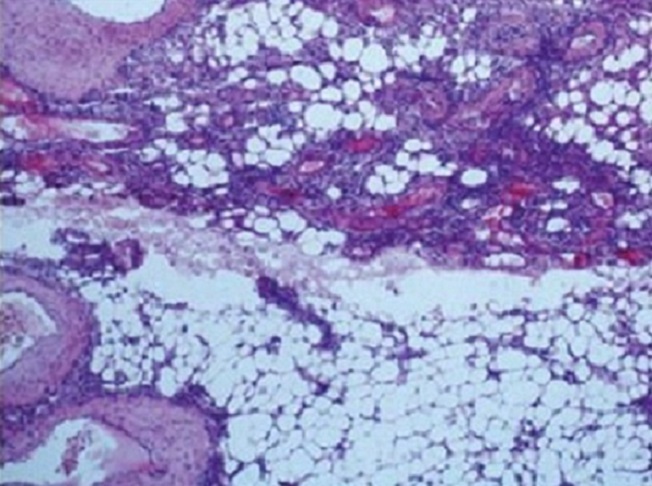
Aspect à la microscopie optique on note le triple contingent adipeux, musculaire lisse et vasculaire

On a opté la surveillance chez une patiente qui présentait une lésion de 42 mm asymptomatique, de découverte fortuite. L'embolisation artérielle sélective a été proposé chez 2 patients qui présentait une AML bilatérale, la première a vu le succès tandis que la seconde s'est présenté aux urgences dans un tableau d'hématurie massive et un hématome rétropéritonéale et une néphrectomie d'hémostase droite a été réalisé. Trois néphrectomies d'hémostase ont été réalisé par voie de lombotomie (Figure 3), une néphrectomie par voie sous costale pour doute diagnostic sur la nature maligne de la masse et deux néphrectomies partielles pour préservation du capital néphronique. Dans notre série on a noté un cas de décès par complication thromboembolique survenant dans le service de réanimation en post opératoire. L’étude histologique a permis de poser le diagnostic d'AML rénal (Figure 4).

## Discussion

L′angiomyolipome (AML) est une tumeur bénigne qui représente 1 à 3% des tumeurs solides du rein. Classiquement, ce type de tumeur s′inscrit dans le cadre de la Sclérose Tubéreuse de Bourneville (STB), qui est une maladie autosomique dominante à pénétrance variable. L′atteinte rénale est alors multiple et bilatérale dans 50 à 80% des cas. Mais, l′AML rénal peut être découvert en dehors de toute phacomatose, il est alors isolé, unilatéral avec une forte prédominance féminine [4, 5]. L'angiomyolipome est classiquement défini comme étant une tumeur triphasique, comportant en proportions variables, un contingent graisseux le plus souvent prédominant, un contingent musculaire lisse et un autre vasculaire [2, 4]. La taille d′un angiomyolipome rénal peut varier de quelques millimètres à 20 cm de diamètre [4].

L′évolution d′une telle lésion est variable dans le temps, le pourcentage de croissance annuel est estimé à 5% en cas de lésion unique, 22% en cas de lésions multiples sans association avec une STB et 18% en association à une STB [5, 6] Le risque évolutif majeur est lié aux complications hémorragiques qui peuvent mettre en jeu le pronostic vital, puisque 20% à 30% des patients sont en choc lors de la rupture [2]. En effet, l'angiomyolipome est la deuxième cause rénale d'hémorragie retro-péritonéale après le carcinome à cellules rénales (CCR) [2–4, 7].

Dans la majorité des cas, l′AML est une tumeur isolée, unique, asymptomatique, découverte de manière fortuite chez la femme, Les manifestations cliniques sont identiques dans les formes sporadiques et dans les formes associées à la STB, la symptomatologie est dominée par les lombalgies, l'hématurie qui peut être microscopique ou macroscopique traduisant la rupture de l'AML dans la voie excrétrice, d'autre signes peuvent être constatés: masse palpable (10 à 20% des cas), état de choc hémorragique qui survient dans 9 à 15% des cas et menace le pronostic vital [2–4, 7, 8], signe inflammatoire (fièvre, hyperleucocytose) [2, 4, 8] et des signes digestives (nausées, vomissement) [7, 8], HTA 7 à 30% des cas et peut disparaitre après l'intervention [7]. Il n'existe pas de marqueur biologique spécifique de l'AML [7, 9].

La tomodensitométrie abdominale s′est imposée comme l′examen de choix dans la détection des tumeurs rénales, elle va permettre dans la grande majorité des cas de poser le diagnostic positif d′AML, sous réserve d′une technique d′examen correcte. Sa sensibilité est proche de 90% en matière de diagnostic des AML [4, 10]. L′objectif est la mise en évidence au sein de la masse rénale d′une composante graisseuse, caractérisée par des densités négatives associées au contingent vasculaire et méiomyomateux, Adison a proposé une classification des AML en 4 types tomodensitométriques [11]: Type-I: essentiellement graisseux (habituellement moins de 2 cm de diamètre et intra rénal): 54%; Type-II: partiellement graisseux (intra-rénal ou bourgeonnant): 29%; Type-III: peu graisseux (plus bourgeonnant et péri-rénal): 11%; Type-IV: sans graisse, peut être petit ou large, intra-rénal ou bourgeonnant, mais toujours homogène et hyperdense: 6%.

Classiquement le traitement des AMLs supérieurs à 4 cm est chirurgical par néphrectomie totale ou partielle; cependant au cours de la dernière décennie de nombreuses alternatives conservatrices ont vu le jour dans le but de préserver l′unité rénale [2, 4, 12]. Les travaux d′Osterling ont popularisé la surveillance active pour les AMLs asymptomatiques et de petite taille, tandis que l′embolisation artérielle sélective concurrence de plus en plus la chirurgie [2]. Les perspectives thérapeutiques d′avenir en matière d'AML sont représentées par les chirurgies mini-ablatives (cryoablation et la radiofréquence), ainsi que par la thérapie ciblée dont les premiers résultats publiés semblent encourageants. La prise en charge des angiomyolipomes n'est pas bien codifiée. OSTERLING a établi des recommandations pour la prise en charge de ce type de lésions qui laissent toutefois, une grande liberté d′initiative à l′urologue traitant [2].

Des tumeurs symptomatiques de 4 cm ou plus volumineuses doivent bénéficier d'une angiographie. En fonction, une embolisation sélective, une tumorectomie ou une néphrectomie partielle doivent être discutées; un volumineux AML asymptomatique, doit être suivi tous les 6 mois par une échographie ou une tomodensitométrie; les lésions symptomatiques de moins de 4 cm doivent être surveillées attentivement et si les symptômes persistent, une embolisation sélective ou un traitement chirurgical conservateur doivent être envisagés; un AML asymptomatique de moins de 4 cm doit être surveillé annuellement avec une échographie ou une tomodensitométrie; enfin, lorsque l'AML est révélé par une hémorragie incontrôlable menaçant le pronostic vital, l'artériographie pratiquée en urgence, permet de localiser et de contrôler le saignement, tout en respectant au maximum le parenchyme rénal fonctionnel. En cas d′échec ou d′indisponibilité de cette technique, la néphrectomie d′hémostase en urgence reste le dernier recours.

## Conclusion

L'angiomyolipome rénal est une tumeur rénale bénigne, quoiqu'il présente une variante plus agressive à potentiel de malignité qui est la forme épithélioïde. Il se présente sous deux formes: l'une sporadique et isolée chez la femme de la cinquième décennie; l'autre en association à une pathologie héréditaire à transmission autosomique dominante: la Sclérose tubéreuse de Bourneville. Le principal risque évolutif de ces tumeurs reste la rupture hémorragique spontanée qui peut menacer le pronostic vital du patient. Le diagnostic d'angiomyolipome repose sur l'examen tomodensitométrique abdominal, qui va permettre la mise en évidence d'une composante graisseuse quasi pathognomonique. Aucun consensus ne régit la prise en charge de cette affection.
